# The Effect of Technique Selection in Labiaplasty Surgery: Analysis of Aesthetic and Functional Outcomes

**DOI:** 10.3390/jcm14248923

**Published:** 2025-12-17

**Authors:** Elif Ucar, Melih Bestel, Burak Huseyin Ucar, Ozan Dogan

**Affiliations:** 1 Faculty of Health Sciences, Department of Midwifery, Istanbul Esenyurt University, 34510 Istanbul, Turkey; 2 Department of Plastic, Reconstructive and Aesthetic Surgery, Prof. Dr. Cemil Tascioglu City Hospital, Health Sciences University, 34384 Istanbul, Turkey; 3 Faculty of Medicine, Department of Gynecology and Obstetrics, Istanbul Nişantaşı University, 34398 Istanbul, Turkey

**Keywords:** labiaplasty, trim resection, wedge resection, FGSIS, FSFI

## Abstract

**Background/Objectives**: This study aimed to compare the effects of two different labiaplasty techniques, trim resection and modified wedge resection, on postoperative patient comfort, complication rates, and sexual function. **Methods**: Between 2021 and 2025, female patients who underwent labiaplasty using two different techniques at a tertiary care centre were retrospectively evaluated. The patients were divided into two equal groups: Group 1 underwent wedge resection, and Group 2 underwent trim resection. Postoperative outcomes were assessed at the first week, first month, and sixth month. Sexual and aesthetic outcomes were evaluated using the Female Sexual Function Index (FSFI) and the Female Genital Self-Image Scale (FGSIS). **Results**: A total of 40 female patients were included in the study. A statistically significant postoperative increase was observed in the total FGSIS and FSFI scores in both groups (*p* < 0.001 for all comparisons). Trim resection resulted in greater improvement in aesthetic satisfaction and body image, as reflected by higher postoperative FGSIS scores. In contrast, wedge resection produced more pronounced improvements in sexual function, particularly in the FSFI subdomains of arousal, orgasm, and satisfaction. Notably, the three patients who developed suture dehiscence at the first postoperative week had all undergone wedge resection and were active smokers. **Conclusions**: Both surgical techniques were effective in enhancing aesthetic satisfaction and sexual function following labiaplasty. While the trim technique appeared superior in improving aesthetic outcomes, the wedge technique provided greater benefits in specific sexual function parameters.

## 1. Introduction

Labiaplasty is a surgical procedure that aims to correct the size, shape and symmetry of the labia, and it has become quite popular in recent years. Social perceptions of beauty, increasing body image-related psychosocial factors and physical discomfort are the main factors that lead women to seek this surgery [1,2], though women may also request these operations to alleviate symptoms of distal vulvar discomfort or recurrent infections associated with asymmetrical or excessively large labium minus tissue [3].

While this surgical procedure primarily addresses aesthetic and some functional issues, studies show that most women seeking labiaplasty are dissatisfied with the appearance of their genitalia, as opposed to having significant functional impairments [4,5]. Labiaplasty is performed using various techniques to address these dissatisfactions, with surgical approaches ranging from the less invasive trim resection technique to more complex procedures involving flap reconstruction or tissue mobilisation, depending on the conditions being addressed [6]. The former, trim resection, is a procedure that aims to minimise postoperative complications, but it can also be used to address aesthetic and functional concerns, as well as such conditions as lichen sclerosus and clitoral phimosis [7,8]. Meanwhile, wedge resection aims to enhance the aesthetic and functional characteristics of the labia minora while preserving sufficient tissue structure. This method involves the excision of a wedge-shaped section of labial tissue, providing a more natural contour compared to traditional straight-line resection techniques. A study highlighting various labiaplasty techniques shows that wedge resection, when performed by experienced surgeons, can provide both cosmetic and functional improvements [9]. Clinical outcomes indicate that patients generally report elevated levels of satisfaction after surgery, with many experiencing higher levels of confidence and comfort compared to before surgery [10].

However, it is crucial to understand the potential risks associated with labiaplasty, as such postoperative complications as wound dehiscence or scarring may occur, particularly in patients with pre-existing conditions, such as lichen sclerosus [3,11]. The adverse effect of comorbid factors on postoperative outcomes has been identified, highlighting the vital importance of careful patient selection [11]. As such, surgeons must provide comprehensive preoperative counselling to patients that covers potential risks and normal anatomical variations, thereby establishing realistic expectations [12]. Furthermore, choosing the appropriate surgical technique is of vital importance to minimising complications, with studies indicating that meticulous attention to preserving the neurovascular supply and the selection of appropriate closure techniques can improve postoperative outcomes [13]. This study aims to determine the effects of different surgical techniques used in labiaplasty on patient complaints and satisfaction, and to evaluate the clinical and demographic factors influencing surgical success.

## 2. Materials and Methods

Between 2021 and 2025, women who underwent labiaplasty using different surgical techniques at a tertiary care center due to dissatisfaction with their labia minora for cosmetic and/or physical reasons were included in the study. The study was planned retrospectively, conducted according to the guidelines of the Declaration of Helsinki, and approved by the Institutional Ethics Committee of Istanbul Esenyurt University (protocol code E-12483425-604.01-64536 and date of approval 9 July 2025).

The patients’ age, obstetric history, and presenting complaints were evaluated. The study included women with labial asymmetry, pain caused by the labia being pulled into the vagina during intercourse, chronic vaginitis, inability to wear tight clothing, itching, personal hygiene problems, dyspareunia, pain during exercise, recurrent urinary tract infections and deviation of urinary flow due to the labia. Women who were to undergo only labiaplasty were included, whereas women who were to undergo additional genital aesthetic procedures, such as vaginoplasty, hoodoplasty, anterior colporrhaphy, posterior colporrhaphy or perineoplasty, were not included in the study.

Patients were retrospectively identified and subsequently allocated into two equal groups: the first 20 patients who underwent wedge resection were assigned to Group 1, and the subsequent 20 patients who underwent trim resection were assigned to Group 2. All procedures were performed by the same experienced specialist (E.U.). In the postoperative period, patients were hospitalised for one night. During hospitalisation, ice packs were applied for 10 min every hour. The same antibiotic therapy and analgesic regimen were administered during hospital stay and continued for one week following discharge. Postoperative follow-up data at 1 week, 1 month, and 6 months—based on available medical records—were reviewed. Postoperative complaints and complications were documented retrospectively. Sexual function was assessed using the validated versions of the Female Genital Self-Image Scale (FGSIS) and the Female Sexual Function Index (FSFI), based on the preoperative and 6-month postoperative records available in the patients’ medical files. All results were documented.

The FGSIS is a scientific measure used to assess women’s perceptions, attitudes and feelings about their own genital regions. It consists of seven items, and the total score ranges from 7 to 28. A high score indicates a more positive genital perception, while a low score indicates anxiety, shame or dissatisfaction with genital appearance [14].

One of the most commonly used scales for assessing female sexual function, the FSFI aims to measure different dimensions of sexuality over the past month. This self-report scale, consisting of 19 items in total, covers six key areas: sexual desire, arousal, vaginal lubrication, orgasm, satisfaction and pain. Each sub-domain is scored using specific questions, and the overall total reflects the woman’s level of sexual function. The higher the score, the better the sexual experience [15].

### 2.1. Surgical Procedure

#### 2.1.1. Trim Resection

**Figure 1:**
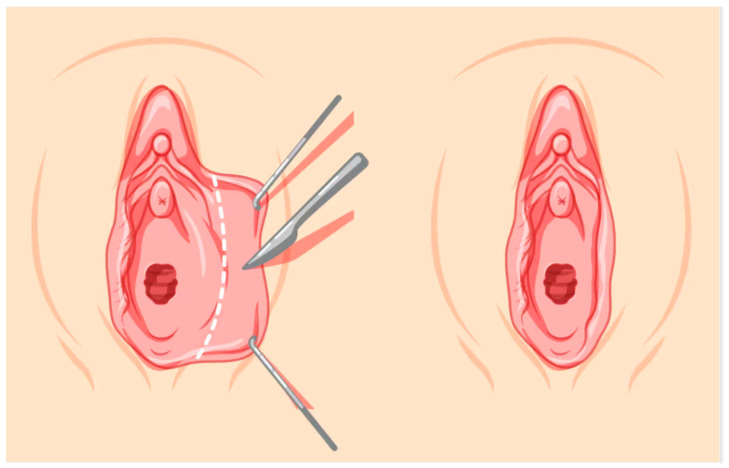
Trim Resection.

**Figure 2:**
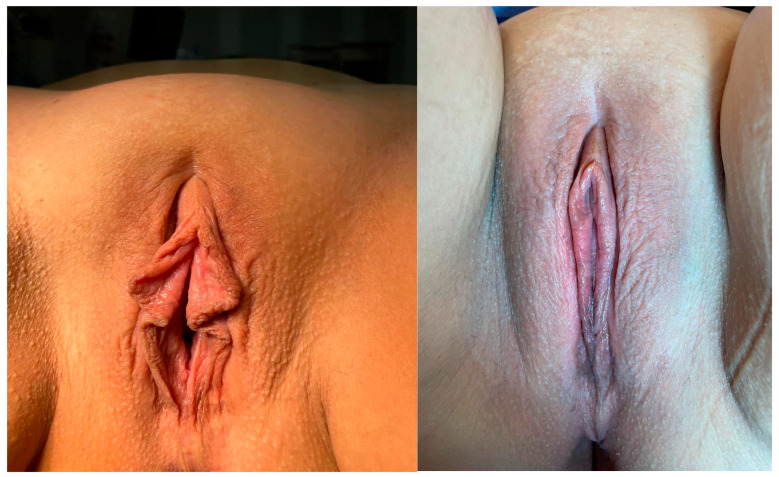
Preoperative and 6 month after operation images of trim resection.

Under spinal anaesthesia and appropriate conditions, the area to be resected was marked with a sterile marker pen from the frenulum to the posterior labium minus. The area to be resected was cut with a scalpel, and resection was performed with scissors. After haemostasis control, the labium minus was sutured individually with 4–0 Vicryl Rapide (Figure 1 and Figure 2).

#### 2.1.2. Modified Wedge Resection

**Figure 3:**
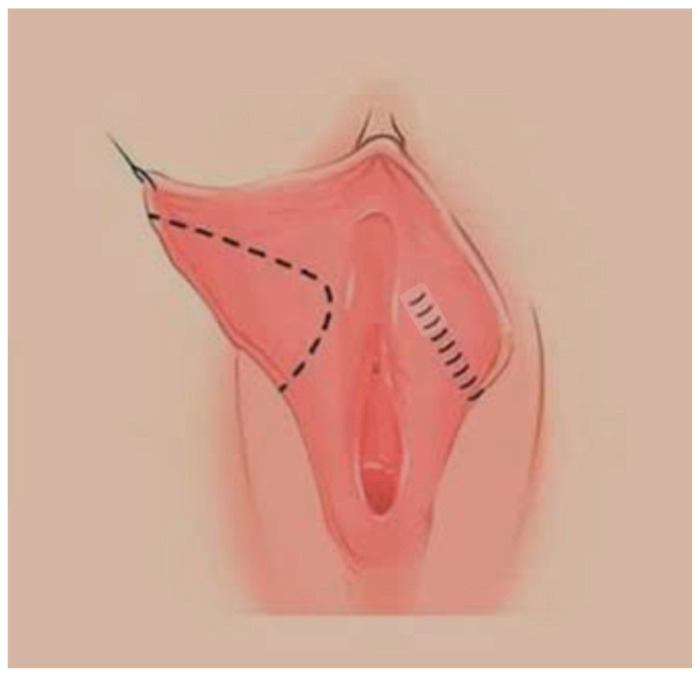
Modified Wedge Resection.

**Figure 4:**
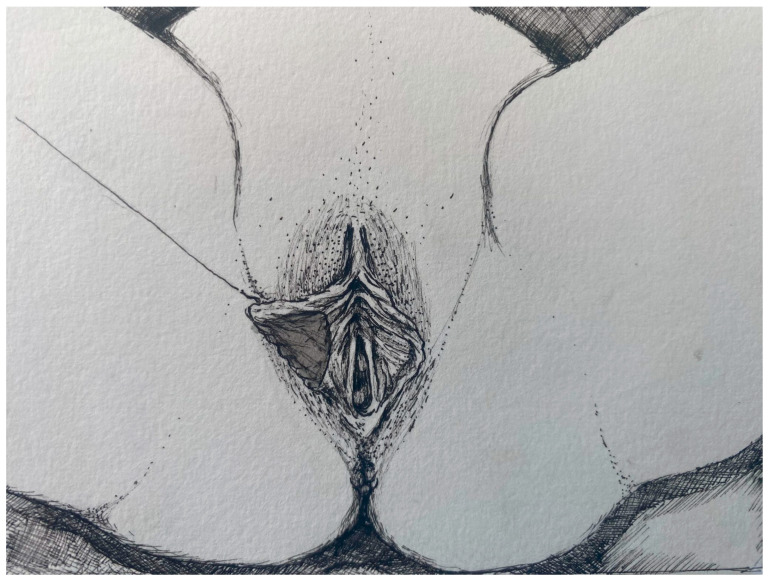
Modified Wedge resection.

**Figure 5:**
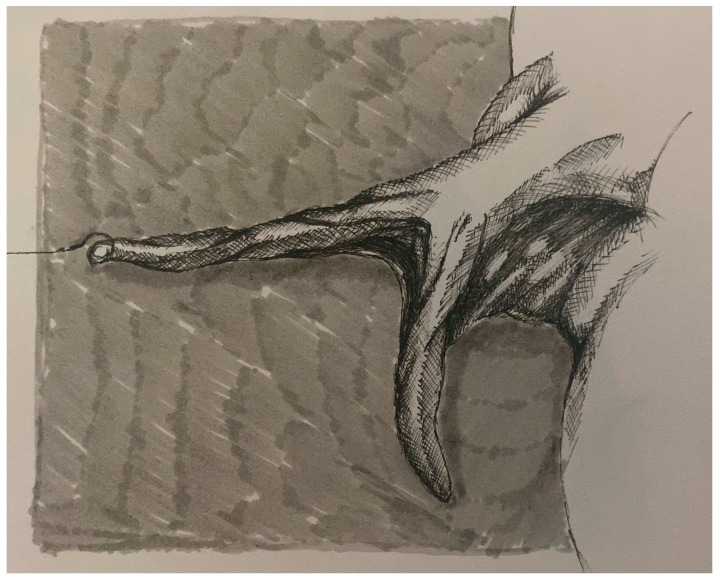
Lateral view of the residual labia minora tissue following resection.

**Figure 6:**
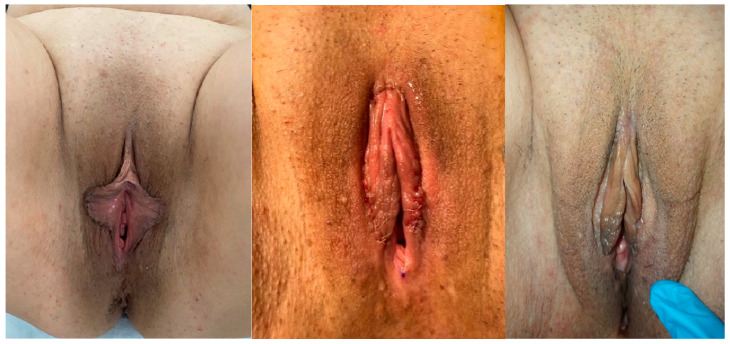
Preoperative, postoperative and 6 months after operation images of wedge resection.

Under spinal anaesthesia and appropriate conditions, the natural contour of the labium was determined from the frenulum to the posterior labium minus. The course of the labial artery was identified using a light source for preservation, and the area to be resected was marked with a sterile marker pen. The area to be resected was cut with a scalpel, and resection was performed with scissors. After haemostasis control, the labium minora was sutured individually with 4–0 Vicryl Rapide (Figure 3, Figure 4, Figure 5 and Figure 6).

### 2.2. Statistical Analysis

All statistical analyses were performed using IBM SPSS Statistics version 24.0 (IBM Corp., Armonk, NY, USA). A *p* value of <0.05 was considered statistically significant for all tests.

Within the scope of descriptive statistics, mean (X^−^), standard deviation (SD), median (M), minimum, and maximum values were calculated for continuous variables, whereas categorical variables were expressed as frequency (n) and percentage (%).

The distribution of continuous variables was assessed using the Shapiro–Wilk test. As most variables did not show normal distribution, non-parametric tests were employed. The Wilcoxon signed-rank test was used for intragroup comparisons of preoperative and postoperative FGSIS and FSFI scores, while the Mann–Whitney U test was used for intergroup comparisons between the wedge and trim techniques.

A *p* value < 0.05 was accepted as the threshold for statistical significance in all analyses.

## 3. Results

The ages of the 40 female patients included in the study ranged from 22 to 50 years, with an average age of 34.8 years in Group 1 and 38.2 years in Group 2 (Table 1).

When complaints were examined, it was observed that the most common reasons for requesting surgery were sexual dissatisfaction and aesthetic concerns. Labial asymmetry was a more frequent reason for surgery in the wedge resection group, while self-image dissatisfaction was more prominent in the trim resection group. Although smoking and complication rates were slightly higher in the wedge group, complication rates were generally low in both techniques (Table 2, Table 3 and Table 4).

A significant increase in total FGSIS and FSFI scores was observed postoperatively in both groups (*p* < 0.001 for all comparisons), though the trim technique led to a greater increase in postoperative FGSIS scores, demonstrating superiority in terms of aesthetics and body image. In contrast, the wedge technique was found to be more advantageous in terms of sexual function, with higher postoperative FSFI scores (Table 5).

Postoperative scores for desire, lubrication, orgasm, satisfaction and pain in the FSFI subcategories increased significantly in both groups (*p* < 0.001) (Table 6).

Notably, the wedge group showed marked increases in arousal, orgasm and satisfaction, while the trim group showed a decrease in pain and a strong improvement in orgasm scores. However, the increase in arousal in the trim group was not statistically significant (*p* = 0.498).

Meanwhile, the wedge resection technique was applied in all three cases where complications developed, and suture dehiscence was observed in these patients. The common feature of these three cases is that they were active smokers.

## 4. Discussion

This study demonstrates that both surgical techniques provide a significant improvement in aesthetic satisfaction and sexual function. However, the trim resection technique was found to be superior, particularly in terms of increased aesthetic satisfaction and reduced pain during sexual intercourse in the postoperative period, while the wedge resection technique provided a more pronounced improvement in sexual function parameters.

Labiaplasty procedures have become increasingly popular surgical interventions in recent years due to rising aesthetic and functional expectations. Sexuality, aesthetic concerns and social expectations play a significant role in women’s assessment of their genital appearance. However, aesthetic or reconstructive improvements in women’s genital appearance are not limited to physical satisfaction; they also create lasting and multidimensional positive effects in the areas of psychosocial and sexual health [16]. Numerous studies in the literature report that these operations have positive effects on women’s body image, self-confidence and quality of sexual life [2,17]. In fact, it has been reported that increased satisfaction with genital appearance is associated with enhanced psychosocial well-being and increased sexual satisfaction [18,19]. Furthermore, studies evaluating the sexual function of women who have undergone labiaplasty and their partners have shown that the surgery increases sexual satisfaction not only for the woman but also for her male partner [20,21]. These findings emphasise the importance of considering both the woman’s and her partner’s expectations in surgical planning; in particular, balancing the extent of resection and mucosal/aesthetic concerns may enable a more positive post-operative sexual life for both individuals. It has been suggested that preserving more than 1 cm of labial tissue leads to more favourable outcomes in terms of sexual function, and therefore, as much labial tissue as possible should be preserved [20]. In addition, it has been observed that the application of different surgical techniques can have varying effects on aesthetic outcomes, complication rates and levels of functional recovery [9].

The trim resection technique is one of the most frequently preferred methods due to its simple surgical applicability and short operation time, aiming to create a smoother, more even and uninterrupted edge by removing excess tissue along the free edge of the labium minora [22]. This technique provides a regular contour to the labial edges and results in a significant reduction in complaints of pain and irritation in the postoperative period [23]. Various series have reported that trim resection achieves a high success rate in terms of aesthetic satisfaction and is particularly effective at reducing concerns about external appearance. In cases with edge irregularities and pronounced hyperpigmentation, the trim resection approach is frequently preferred to enhance visual integrity [24]. In addition, long-term improvements in sexual satisfaction have been observed. However, such complications as scar tissue development at the incision line, edge asymmetry or sensory changes may occur due to the alteration of the natural mucosal edge. Long-term literature reviews report that genital appearance scores and aesthetic satisfaction improve permanently after labiaplasty, regardless of the technique used, while trim resection is considered to optimise visual results in selected patient profiles, with the aim of making the margin line ‘smoother’ [25]. Therefore, trim resection may be considered a favourable option, particularly in patient groups focused on aesthetic appearance and symptomatic relief. In our study, we found that aesthetic satisfaction was higher with the trim resection technique compared to the wedge resection technique, and we observed lower rates of pain complaints during sexual intercourse in the postoperative period, findings that are consistent with the literature.

Conversely, the wedge resection technique offers an approach that preserves anatomical integrity to a greater extent and maintains the natural mucosal margin. The most significant advantage of this method is that it improves not only aesthetic appearance but also the functional outcomes. Indeed, the literature shows that there are significant increases in sexual function scores after wedge resection; for instance, more positive results were obtained in terms of sexual satisfaction, orgasm and desire levels compared to the trim technique [18,23]. In addition, wedge resection provides a more natural appearance and long-term aesthetic results thanks to the preservation of edge integrity. Zahedi et al. emphasise that surgeons should consider functional expectations in addition to aesthetic concerns when choosing between the trim or wedge techniques based on patient anatomy, though they suggest the wedge technique may have a higher potential to improve functional outcomes, depending on such variables as the amount of residual tissue based on patient anatomy [24]. One review also states that both techniques increase aesthetic satisfaction, but the wedge technique may offer advantages in terms of ‘labial wedge’ and tissue preservation, emphasising that the latter is an important factor in long-term sexual and functional satisfaction [26]. In line with this finding, the preservation of more labial tissue in the wedge resection compared to the trim resection technique can be considered a factor allowing greater improvement in sexual function. These findings support the preference for wedge resection, particularly in terms of sexual function. In our study, we also observed greater improvements in sexual function parameters [particularly arousal and satisfaction) with the wedge resection technique compared to the trim resection technique, a finding that is consistent with the literature.

One study indicates that failure to pay attention to the course of the labial vascular structure may compromise mucosal integrity and negatively affect wound healing [27]. Therefore, particular care should be taken to preserve the labial artery tracing during surgery [28]. Furthermore, as the use of nicotine, cocaine or other vasoconstrictor agents might potentially compromise wound healing and increase risk of suture-line separation—therefore caution is advised in patients with such risk factors [29]. In this study, suture line separation developed in the postoperative period in three cases where wedge resection was performed. This complication may be due to depressurisation resulting from accidental damage to the labial vessels, which run immediately beneath the mucosa anatomically. The fact that all three cases were smokers may have increased the risk of complications.

The primary strength of this study lies in the comparative evaluation of two distinct surgical techniques using two validated questionnaires, allowing for a comprehensive assessment of both aesthetic outcomes and functional improvements following surgery. However, several limitations should be acknowledged. The relatively small sample size may reduce the statistical power and limit the generalizability of the findings. In addition, the retrospective design inherently constrains causal inference and may introduce selection or information bias. Furthermore, no dedicated psychological assessment questionnaire was administered to evaluate the psychological status of the patients included in the study. Furthermore, no statistical adjustments were made for potential confounding variables such as age, smoking status, parity, or preoperative sexual activity levels, which could have influenced the observed results. The relatively short follow-up period also limits the evaluation of long-term complications and recurrence. Future prospective studies with larger cohorts, extended follow-up durations, and multivariate analyses controlling for confounding factors are warranted to yield a more robust understanding of the aesthetic and functional outcomes associated with both techniques.

## 5. Conclusions

Both surgical techniques applied for labial reconstruction were found to result in significant improvements in aesthetic satisfaction and sexual function. Although the trim resection technique appeared to be more advantageous in terms of aesthetic satisfaction and reduction in pain during postoperative sexual intercourse, the wedge resection technique provided more pronounced improvements in the subdomains of sexual function, particularly arousal and satisfaction. These findings suggest that the amount of residual labia minora tissue is a critical determinant of sexual function, and we propose that this factor should not be overlooked during surgical planning. Furthermore, the observation that suture dehiscence in the wedge group occurred exclusively in smokers indicates that smoking may adversely affect wound healing and increase the risk of complications. Therefore, when selecting a surgical technique for labiaplasty, the patient’s primary expectations should be carefully evaluated, and the choice of technique should be tailored accordingly. In addition, comprehensive assessment of comorbid factors, such as smoking, is strongly recommended.

Future prospective, multicentre studies with larger sample sizes, longer follow-up durations, and comprehensive multivariate analyses are warranted to further clarify the long-term aesthetic and functional impacts of different labiaplasty techniques and to establish more robust evidence-based guidelines for surgical technique selection.

## Figures and Tables

**Table 1:** Demographic data.

Variables	Wedge Resection Min–Max	Wedge Resection Median ± SS	Trim Resection Min–Max	Trim Resection Median ± SS
**Age**	22–48	34.8 ± 7.3	30–50	38.2 ± 6.2
**Gravida**	0–6	2.0 ± 1.6	0–5	2.0 ± 1.0
**Parity**	0–6	2.0 ± 1.7	0–5	2.0 ± 1.0

**Table 2:** Distribution of complaints by group.

	Wedge Resection	Trim Resection
**Discharge (poor hygiene)**	4 (20.0%)	2 (10.0%)
**Decreased sexual satisfaction**	7 (35.0%)	3 (15.0%)
**Dyspareunia**	3 (15.0%)	4 (20.0%)
**Aesthetic appearance**	2 (10.0%)	0 (0.0%)
**Low self-esteem**	4 (20.0%)	11 (55.0%)

**Table 3:** Reason for requesting surgery by group.

	Wedge Resection	Trim Resection
**Low self-esteem**	4 (20.0%)	11 (55.0%)
**Labial asymmetry**	13 (65.0%)	4 (20.0%)
**Partner request**	3 (15.0%)	5 (25.0%)

**Table 4:** Smoking prevalence and complication rates by group.

Smoking	Wedge Resection	Trim Resection
**Yes**	3 (15.0%)	5 (25.0%)
**No**	17 (85.0%)	15 (75.0%)
**Complications**		
**Suture dehiscence**	3 (15.0%)	0 (0.0%)
**None**	17 (85.0%)	20 (100.0%)

**Table 5:** FGSIS and FSFI Total Scores.

	Preoperative FGSIS Average	Postoperative FGSIS Average	*p* (FGSIS)	Preoperative FSFI Average	Postoperative FSFI Average	*p* * (FSFI)
**Wedge Resection**	11	22	**<0.001**	24	33	**<0.001**
**Trim Resection**	10	26	**<0.001**	22	32	**<0.001**

* Wilcoxon signed-rank test.

**Table 6:** FSFI Subcategories.

	Subcategories	Preoperative Average	Postoperative Average	*p* *
**Wedge Resection**	Desire	4.1	5.0	<0.001 *
**Wedge Resection**	Arousal	4.4	5.5	<0.001 *
**Wedge Resection**	Lubrication	4.4	5.0	<0.001 *
**Wedge Resection**	Orgasm	4.6	6.0	<0.001 *
**Wedge Resection**	Satisfaction	3.9	5.8	<0.001 *
**Wedge Resection**	Pain	2.4	6.0	<0.001 *
**Trim Resection**	Desire	4.1	4.9	<0.001 *
**Trim Resection**	Arousal	4.9	5.0	0.498
**Trim Resection**	Lubrication	3.9	4.9	<0.001 *
**Trim Resection**	Orgasm	3.6	5.8	<0.001 *
**Trim Resection**	Satisfaction	3.2	5.6	<0.001 *
**Trim Resection**	Pain	2.4	6.0	<0.001 *

* Wilcoxon signed-rank test.

## Data Availability

The original contributions presented in this study are included in the article/Supplementary Materials. Further inquiries can be directed to the corresponding author.

## Supplementary Materials

The following supporting information can be downloaded at: https://www.mdpi.com/article/10.3390/jcm14248923/s1, STROBE Statement—checklist of items that should be included in reports of observational studies.
